# Assessment of Hip Fracture Risk Using Cross-Section Strain Energy Determined by QCT-Based Finite Element Modeling

**DOI:** 10.1155/2015/413839

**Published:** 2015-10-25

**Authors:** Hossein Kheirollahi, Yunhua Luo

**Affiliations:** ^1^Department of Mechanical Engineering, Faculty of Engineering, University of Manitoba, Winnipeg, MB, Canada R3T 5V6; ^2^Department of Anatomy, Southern Medical University, Guangzhou 510515, China

## Abstract

Accurate assessment of hip fracture risk is very important to prevent hip fracture and to monitor the effect of a treatment. A subject-specific QCT-based finite element model was constructed to assess hip fracture risk at the critical locations of femur during the single-leg stance and the sideways fall. The aim of this study was to improve the prediction of hip fracture risk by introducing a novel failure criterion to more accurately describe bone failure mechanism. Hip fracture risk index was defined using cross-section strain energy, which is able to integrate information of stresses, strains, and material properties affecting bone failure. It was found that the femoral neck and the intertrochanteric region have higher fracture risk than other parts of the femur, probably owing to the larger content of cancellous bone in these regions. The study results also suggested that women are more prone to hip fracture than men. The findings in this study have a good agreement with those clinical observations reported in the literature. The proposed hip fracture risk index based on strain energy has the potential of more accurate assessment of hip fracture risk. However, experimental validation should be conducted before its clinical applications.

## 1. Introduction

Two of the major determinants of proximal femur fractures among the elderly are osteoporosis and sideways fall. The most common serious injury associated with the fall of an elderly person is hip fracture. Furthermore, hip fracture is associated with an up to 20% chance of death, a 25% chance of long term institutionalization, and less than a 50% chance of full recovery [1]. The total number of hip fractures in men and women in 1990 was estimated to be 338,000 and 917,000, respectively, over the world [2]. Assuming no change in the age- and sex-specific incidence, the number of hip fractures is estimated to approximately double to 2.6 million by the year 2025 and 4.5 million by the year 2050 over the world [2]. By the increasing trend in hip fractures because of the aging of the population, the worldwide annual costs of hip fractures in the year 2050 have been estimated to be $131.5 billion [3]. So, hip fracture risk should be assessed in individuals who are at a risk for an osteoporotic hip fracture to provide proper plans to prevent future probable fractures.

Statistical models and bone mineral density (BMD) captured by Dual-Energy X-ray Absorptiometry (DXA) are used for in vivo osteoporotic fracture risk assessment [4, 5]. However, their accuracy in assessment of fractures is limited; most osteoporotic fractures actually occur with BMD measurements that are above the conventional osteoporotic threshold [6]. Fracture Risk Assessment Tool (FRAX) is a tool to evaluate an individual's fracture probability in the next 10 years, adopted by the WHO in 2008 [7]. FRAX does not take into account fall-induced impact force that is critically important in the hip fracture risk assessment [8, 9]. The main limitations of the FRAX include the following: it is a statistical model and fracture risk is not consistent within 10 years with some of the treatment results [10]. Hip structure analysis (HSA) program is now commercially available and is used to automatically assess the geometric and structural parameters of the femur. Whereas HSA is based on a beam model, the deformation of bone is oversimplified, especially in the femoral neck and the intertrochanteric region where osteoporotic femoral fractures most often occur; therefore the deformation is too complicated to be described as a beam model [11].

Finite element models constructed from quantitative computed tomography (QCT) are very helpful in assessing hip fracture risk, as they are based on well-established biomechanical principles and theories. In QCT-based finite element modeling, choosing a proper failure criterion is very important to accurate assessment of hip fracture risk. Commonly used bone failure criteria include von Mises stress and strain criteria [12–15], maximum principle stress and strain criteria [16–19], maximum shear stress criterion [20], and maximum distortion energy criterion [20]. The femur consists of inhomogeneous (porous) cancellous bone and nearly homogenous cortical bone, and their failure mechanisms are different due to their different microstructure. Failure mechanism of the cancellous bone is mostly in the form of spicule (trabecula) buckling, and the failure of denser cancellous bone and the cortical bone is mostly characterized by local cracking [21, 22]. Cortical and cancellous bone have different properties. Cortical bone is usually more brittle [23], while cancellous bone is more ductile. The current failure criteria are not convenient in describing their properties. Therefore, the total strain energy, which integrates all information of stress, strain, and material properties, is a better option [21]. Mirzaei et al. [21, 24] predicted failure strength and failure patterns of human proximal femur and human vertebrae using the strain energy criterion with a QCT-based FE model. Their predictions of the failure loads and failure locations were in a good agreement with the experimental findings. The strain energy criterion is widely used in fracture analysis of engineering materials. It is usually used in crack problems [25–27], composite laminates [28, 29], and bone cement analysis [30]. Therefore, computation of hip fracture risk index (FRI) over a region of interest (ROI) based on the strain energy criterion theoretically should be more accurate for assessing hip fracture risk. To the best of our knowledge, there are currently no published studies that use the strain energy criterion for the hip fracture risk assessment. The objective of this study is to improve the hip fracture risk assessment procedure previously developed by Luo et al. [11] by introducing the strain energy criterion.

## 2. Materials and Methods

**Figure 1:**
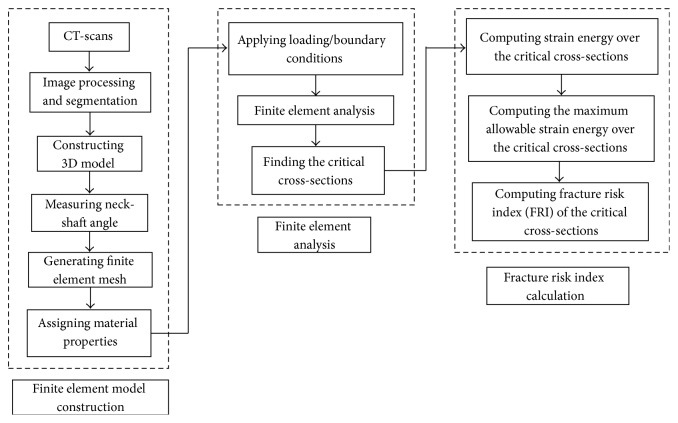
The proposed methodology for calculating hip fracture risk index using the strain energy criterion.

The proposed methodology for assessment of hip fracture risk in the critical regions of femur using the strain energy criterion determined from QCT-based finite element model is shown in Figure 1. The procedure is explained in detail in the following.

### 2.1. QCT-Based Finite Element Model

#### 2.1.1. QCT-Scan of Femur

**Figure 2:**
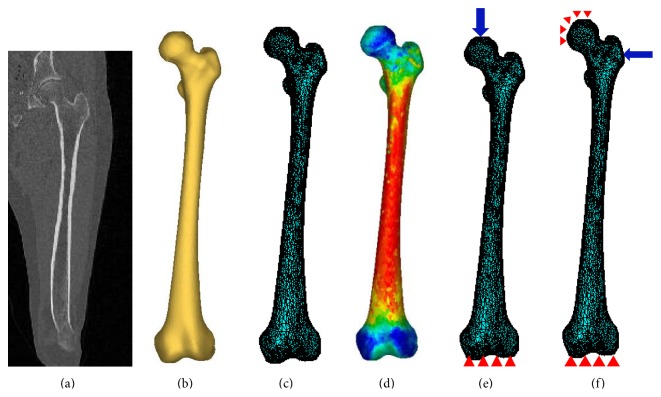
QCT-based finite element modeling: (a) QCT-scan of the subject's femur; (b) 3D model generated from the QCT images; (c) 3D finite element model; (d) distribution of elasticity modulus; (e) single-leg stance configuration; and (f) sideways fall configuration.

The purpose of this study is to accurately assess hip fracture risk, so, a 3D finite element model of subject's femur is required to achieve it. The 3D model can be constructed from the subject's femur QCT images. QCT slices are produced using multiple scanners with a set of proper acquisition and reconstruction parameters (Figure 2(a)). Slice thickness of 1 mm is commonly used. The scanned QCT images are stored in the format of Digital Imaging and Communications in Medicine (DICOM), which can be used for the construction of a 3D FE model. A proper segmentation is done to separate the femur for constructing the 3D model. Each voxel in the QCT-scan has an intensity (or grey scale) that is expressed as Hounsfield Unit (HU), which is correlated to bone density [31, 32]. Threshold value for the cancellous bone is from 100 HU to 200 HU and for the cortical bone is between 200 HU and 2000 HU. QCT images of 60 clinical cases (30 females and 30 males) were acquired from the Winnipeg Health Science Centre in an anonymous way under a human research ethics approval. The subjects are in the age scope from 50 to 82 years (average of 65 years). Statistical information of the clinical cases is listed in Table 1.

#### 2.1.2. Generation of Finite Element Mesh

In the first step, the geometrical model of the femur is generated from clinical QCT images using Mimics (Materialise, Leuven, Belgium). QCT images (in DICOM format) are imported to Mimics for segmentation (Figure 2(a)) and construction of 3D geometric model of the femur (Figure 2(b)). With the 3D geometric model, a FE mesh is generated using the 3-matic module in Mimics (Figure 2(c)). The 4-node linear tetrahedral element SOLID72 in ANSYS was used in this study. To investigate model convergence, FE models with different maximum element edge lengths were created. For each FE model, displacement was calculated under the same loading and boundary conditions. The maximum element edge length that produced converged finite element solutions was obtained and used in all the rest of FE simulations. The number of elements for each case is assigned based on the maximum element edge length that provided converged FE solution, meaning that the number of elements is different from case to case.

#### 2.1.3. Assignment of Material Properties

To construct a more faithful FE model, bone material properties are considered inhomogeneous and isotropic in this study. Information on the inhomogeneous isotropic mechanical properties of the bone can be derived from the CT data using a mathematical relationship between the CT numbers and the mechanical properties of bone. The following empirical equation was used to determine bone ash density (*ρ*_ash_) from HU number [33, 34]:

(1) $$\begin{array}{c} \rho_{\text{ash}}=0.04162+0.000854\;\text{HU}\;\left(\text{g}/{\text{cm}}^{\text{3}}\right). \end{array}$$

Equations (2) and (3), derived by Keller [35], were, respectively, used to assign Young's modulus (*E*) and yield stress (*σ*_*Y*_) according to the bone ash density:

(2) E=10500ρash2.29MPa

(3) σY=116ρash2.03MPa.

A constant Poisson's ratio (*ν* = 0.4) was considered as suggested in the literature [13, 36, 37]. To assign material properties, elements are grouped into a number of discrete material bins using Mimics (Materialise, Leuven, Belgium), to approximately represent the continuous distribution of the inhomogeneous bone mechanical properties. To determine the maximum number of material bins, convergence study was performed. Models with different material bins were created for convergence study. For each FE model, displacement was calculated under the same loading and boundary conditions. The maximum number of material bins that generated converged finite element solutions was obtained.  Figure 2(d) shows the isotropic inhomogeneous distribution of material properties.

### 2.2. Finite Element Analysis Using ANSYS

The finite element model of femur with the assigned material properties was output from Mimics and then imported to ANSYS for finite element analysis. For a precise assessment of hip fracture risk during the single-leg stance and the sideways fall, loading and boundary conditions simulating the single-leg stance and the sideways fall configurations are required in the FE model. To simulate the single-leg stance configuration, 2.5 times the patient's body weight was applied as a distributed load on the femoral head in direction of femoral shaft axis [38] and femur was fixed at the distal end [13, 39]; see Figure 2(e). Consider

(4) $$\begin{array}{c} F_{\text{Stance}}=2.5w\;\left(\text{N}\right), \end{array}$$

where *w* is the subject's body weight in Newton (N). To simulate sideways fall, the distal end of femur was completely fixed and the surface of femoral head was fixed in the loading direction (Figure 2(f)) [40, 41]. The impact force during the sideways fall acting on the greater trochanter perpendicularly (Figure 2(f)) is given by [38, 42]:

(5) $$\begin{array}{c} F_{\text{Impact}}=8.25w{\left(\;\frac{h}{170}\;\right)}^{1/2}\;\left(\text{N}\right), \end{array}$$

where *h* is the height of the subject in centimeter (cm). Loading and boundary conditions on the greater trochanter, the femoral head, and the distal end of femur were applied to a group of nodes using APDL codes (Figures 2(e) and 2(f)). After importing the QCT-based FE model and applying the loading and boundary conditions, finite element analysis was performed and finite element solutions were obtained. In all the analysis, the nodal displacements, stresses, and strains were obtained for each subject.

### 2.3. Detection of the Three Critical Cross-Sections on the Femur

**Figure 3:**
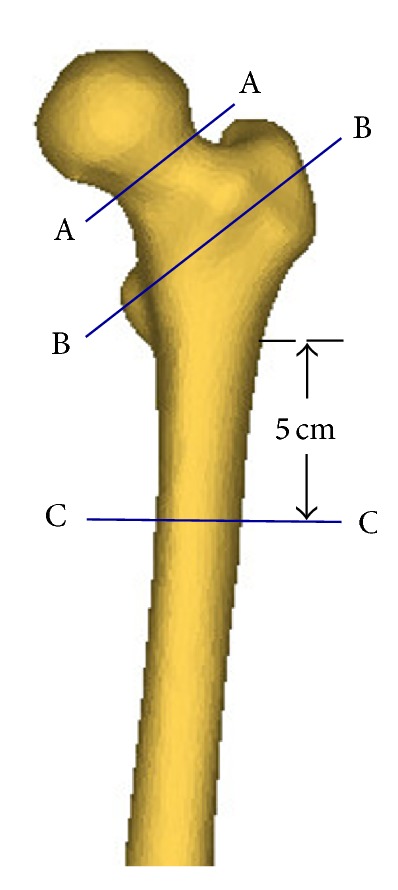
Three critical cross-sections of femur: the smallest femoral neck cross-section (A-A), the intertrochanteric cross-section (B-B), and the subtrochanteric cross-section (C-C).

Hip fractures usually occur at one of the anatomical locations: the femoral neck, the intertrochanter, and the subtrochanter as illustrated in Figure 3. According to clinical observations, 49 percent of hip fractures are intertrochanteric, 37 percent are at femoral neck, and 14 percent are subtrochanteric [43]. Therefore, the smallest femoral neck cross-section (SFN CS), the intertrochanteric cross-section (IntT CS), and the subtrochanteric cross-section (SubT CS) are the three critical cross-sections of femur that usually have the highest fracture risk (Figure 3). To determine the smallest femoral neck cross-section and the intertrochanteric cross-section, neck-shaft angle is needed. The neck-shaft angle is the angle between the femoral neck axis and the femoral shaft axis. This angle traditionally is measured on conventional radiography images, or using 2D images projected from CT/MRI data. In spite of their popularity, these methods are based on oversimplification of the real 3D anatomy and may lead to large errors due to the inaccuracies in selection of the measurement plane [44–46]. In this study, the neck-shaft angle was measured using a 3D measurement technique based on fitting functions. In this technique, the shapes of particular parts of the femur are approximated using geometric entities such as circle, cylinder, and sphere, which are well fitted to the actual anatomy, and the geometrical relationships between these entities are obtained to estimate the neck-shaft angle.

**Figure 4:**
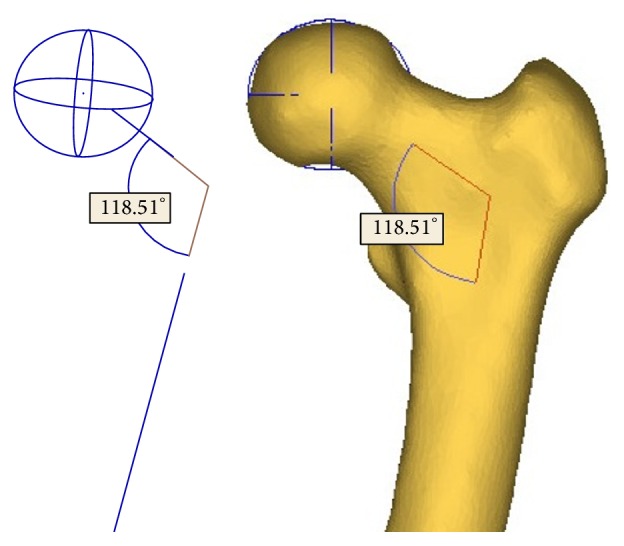
Neck-shaft angle measured by the fitting functions in the 3-matic module of Mimics.

First, a sphere was fitted to the femoral head, to obtain the position of the joint's centre of rotation, which is also the femoral head centre. Then, the femoral neck axis and the femoral shaft axis are identified by applying the “fit ruled surface direction” function on the femoral neck and shaft. All fitting functions were applied using the 3-matic module in Mimics. The neck-shaft angle was also measured by 3-matic module of Mimics (Figure 4). With the femoral neck-shaft angle, the intertrochanteric cross-section and the smallest femoral neck cross-section were found using in-house computer codes. The smallest femoral neck cross-section is chosen as the cross-section with the smallest area in the neck region and the intertrochanteric cross-section is chosen as the cross-section that has the largest area in the intertrochanteric region [47]. By using APDL codes, perpendicular planes on the femoral neck axis were determined and then areas of the cross-sections were calculated. The planes with the smallest and the largest areas were chosen, respectively, as the smallest femoral neck cross-section and the intertrochanteric cross-section. The subtrochanteric cross-section is considered five centimeters below the lesser trochanter [48] (Figure 3).

### 2.4. Hip Fracture Risk Index Definition Using the Strain Energy Criterion

**Figure 5:**
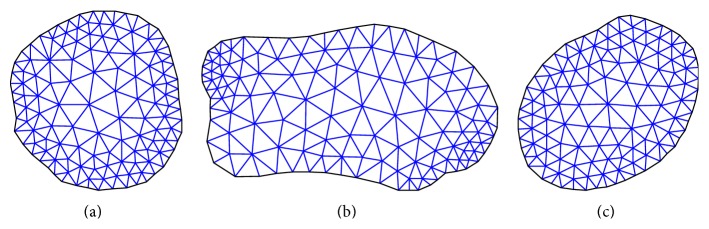
Generated triangle elements over (a) the smallest femoral neck cross-section, (b) the intertrochanteric cross-section, and (c) the subtrochanteric cross-section.

Based on the previous discussion of the bone failure mechanism and microstructure, the strain energy criterion is theoretically more favourable for hip fracture risk assessment. The strain energy at the three critical cross-sections of femur induced by the applied forces was computed using in-house developed MATLAB codes and the data extracted by APDL codes from the obtained finite element solutions. The plane boundaries of the three critical cross-sections, extracted from the finite element mesh, were imported to MATALB to generate a 2D mesh for calculating the cross-section strain energy.  Figure 5 shows the generated triangle elements over the smallest femoral neck cross-section, the intertrochanteric cross-section, and the subtrochanteric cross-section.

The strain energy at the three critical cross-sections induced by the applied forces is the sum of strain energy in all the triangle elements; that is,

(6) $$\begin{array}{c} U=\overset{m}{\underset{i=1}{\sum }}U_{e}, \end{array}$$

where *U*_*e*_ is the strain energy in Element *e* induced by the applied forces and *m* is the number of triangle elements created over the concerned cross-section. Gaussian integration method was used to calculate the strain energy in elements. Integration points in each triangle element were determined using in-house MATLAB codes. By using Gaussian integration method, the strain energy of Element *e* induced by the applied forces is calculated as

(7) $$\begin{array}{c} U_{e}=\overset{\;}{\underset{\;}{\iint }}{\hat{U}}_{e}\;dA\approx \overset{n}{\underset{i=1}{\sum }}W_{i}\left|\;J\;\right|{\hat{U}}_{i}, \end{array}$$

where ${\hat{U}}_{e}$ is the strain energy density of Element *e*; ${\hat{U}}_{i}$ is the strain energy density at the integration point *i* of in Element *e*; *W*_*i*_ is the weight at the integration point; |*J*| is the determinant of the Jacobean matrix of the triangle element; and *n* is the number of integration points over the triangle element. The strain energy density at an integration point (*i*) was determined from the finite element solutions obtained by the QCT-based FE model; that is,

(8) $$\begin{array}{c} {\hat{U}}_{i}=\frac{1}{2}{\left\{\;\sigma \;\right\}}^{T}\left\{\;\varepsilon \;\right\}, \end{array}$$

where {*σ*} = [*D*]{*ε*} and {*ε*} = [*B*]{*d*}. The strain energy density at each integration point can be expressed by the finite element solutions as

(9) $$\begin{array}{c} {\hat{U}}_{i}=\frac{1}{2}{\left\{\;d\;\right\}}_{e}^{T}{\left[\;B\;\right]}_{e}^{T}{\left[\;D\;\right]}_{e}{\left[\;B\;\right]}_{e}{\left\{\;d\;\right\}}_{e}, \end{array}$$

where {*d*} is the displacement vector consisting of displacements at the nodes of Element *e*; matrix [*B*] consists of the derivatives of shape functions of the element; and [*D*] is the material property matrix. Consider

(10) De=E1+ν1−2ν·1−ννν000ν1−νν000νν1−ν00000012−ν00000012−ν00000012−ν,

where Poisson's ratio is constant (*ν* = 0.4) and Young's modulus is a function of the bone density as given in (2). For each integration point, its Young's modulus is determined by the bone density at the point.

The maximum allowable strain energy over a critical cross-section of the femur was also computed from the obtained finite element solutions using in-house MATLAB codes. The maximum allowable strain energy (or the yield strain energy) over a critical cross-section is obtained as

(11) $$\begin{array}{c} U_{Y}=\overset{m}{\underset{i=1}{\sum }}U_{Y}^{e}, \end{array}$$

where *U*_*Y*_^*e*^ is the yield strain energy in Element *e* and *m* is the number of triangle elements over the concerned cross-section. The Gaussian integration method was also used to calculate the maximum allowable strain energy in each triangle element. The maximum allowable strain energy that a triangle element (*e*) can sustain is given by

(12) $$\begin{array}{c} U_{Y}^{e}=\overset{\;}{\underset{\;}{\iint }}{\hat{U}}_{Y}^{e}\;dA\approx \overset{n}{\underset{i=1}{\sum }}W_{i}\left|\;J\;\right|{\hat{U}}_{Yi}, \end{array}$$

where ${\hat{U}}_{Y}^{e}$ is the yield strain energy density in Element *e*; *n* is the number of integration points; and ${\hat{U}}_{Yi}$ is the yield strain energy density at integration point *i* and is calculated as

(13) $$\begin{array}{c} {\hat{U}}_{Yi}=\frac{1}{2}\sigma_{Yi}\varepsilon_{Yi}=\frac{\sigma_{Yi}^{2}}{2E_{i}}, \end{array}$$

where *σ*_*Yi*_ and *E*_*i*_ are, respectively, the yield stress and Young's modulus at the integration point. Both of them are function of bone density, as given in (2) and (3).

Hip fracture risk index (*η*) at a critical cross-section is defined as

(14) $$\begin{array}{c} \eta =\frac{U}{U_{Y}}, \end{array}$$

where *U* and *U*_*Y*_ are, respectively, obtained from (6) and (11).

## 3. Results

### 3.1. Convergence Studies

#### 3.1.1. Element Size in Femur Finite Element Analysis

**Figure 6:**
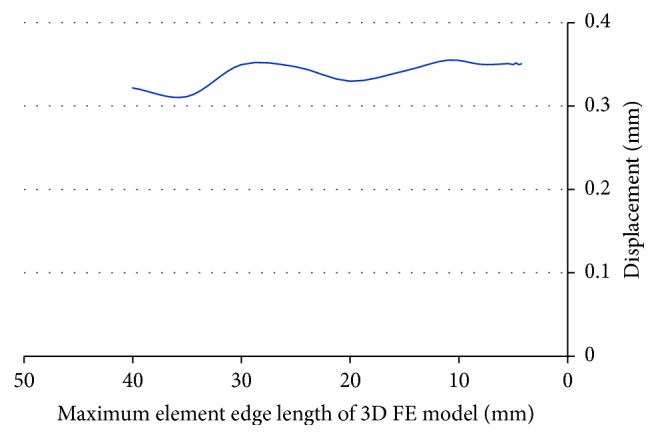
Convergence of finite element solutions with element size.

The convergence of finite element solutions in a representative case is shown in Figure 6. The convergence study showed that the finite element displacements converged with the maximum element edge length smaller than 8 mm. Therefore, in construction of the rest of femur FE models, the maximum element edge length was set to 8 mm.

#### 3.1.2. Assignment of Inhomogeneous Material Properties

For convergence study in assigning the inhomogeneous material properties, 3D femur FE models with different material bins were created. For each FE model with different material bins, the maximum displacement at the smallest femoral neck cross-section was monitored under the same loading and boundary conditions. Data on the displacement were compared among the FE models with different material bins. The results of the convergence study showed that the displacement did not change significantly with the number of material bins larger than 50. Therefore, in the assignment of material properties for all the cases, 50 discrete material bins were considered.

#### 3.1.3. Element Size in Calculating Cross-Section Strain Energy

**Figure 7:**
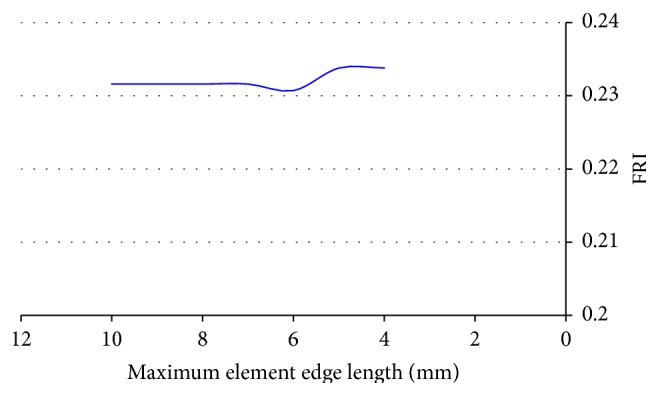
Convergence of FRI.

Convergence study was also performed to determine the element size used in integrating cross-section strain energy, as it affects the calculated fracture risk index (FRI). The FRI at the concerned cross-section was calculated with different maximum element edge lengths. The results are plotted in Figure 7. The FRI did not change significantly with the maximum element edge length smaller than 5 mm. Therefore, the maximum element edge length was set to 5 mm in calculating cross-section strain energy.

#### 3.1.4. The Number of Integration Points in Calculating Cross-Section Strain Energy

The effect of the number of integration points on the calculated FRI was investigated. FRI at the smallest femoral neck cross-section was computed for 5 clinical cases with different number of integration points. The relative errors between FRIs obtained with 3 and 7 integration points are shown in Table 2. As it can be seen, the errors are not significant. Therefore, the 3-point integration rule was used in this study to reduce computational time.

### 3.2. Stress and Strain Patterns at the Three Critical Cross-Sections

**Figure 8:**
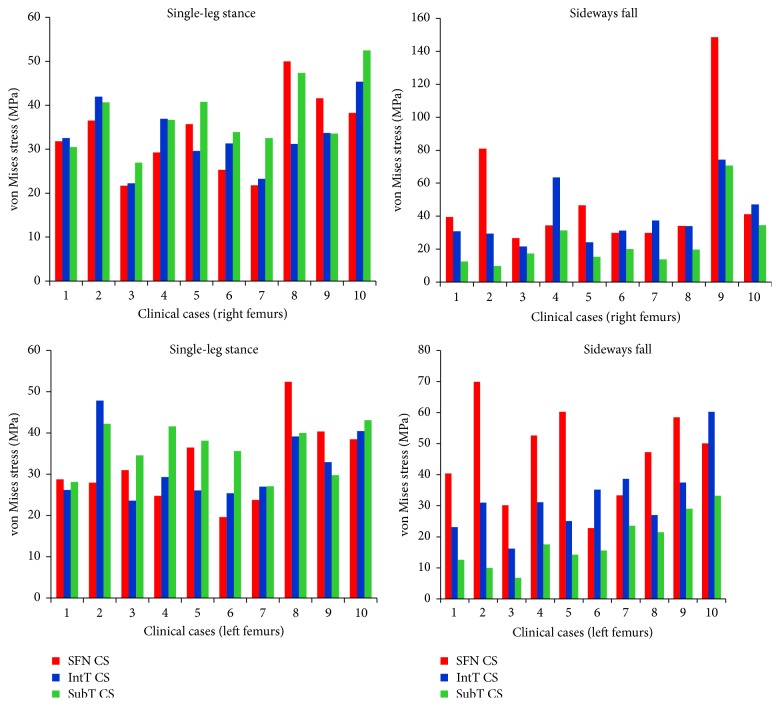
The maximum von Mises stress (Mpa) at the smallest femoral neck cross-section (SFN CS), the intertrochanteric cross-section (IntT CS), and the subtrochanteric cross-section (SubT CS) of right and left femurs of 10 clinical cases during the single-leg stance and the sideways fall.

**Figure 9:**
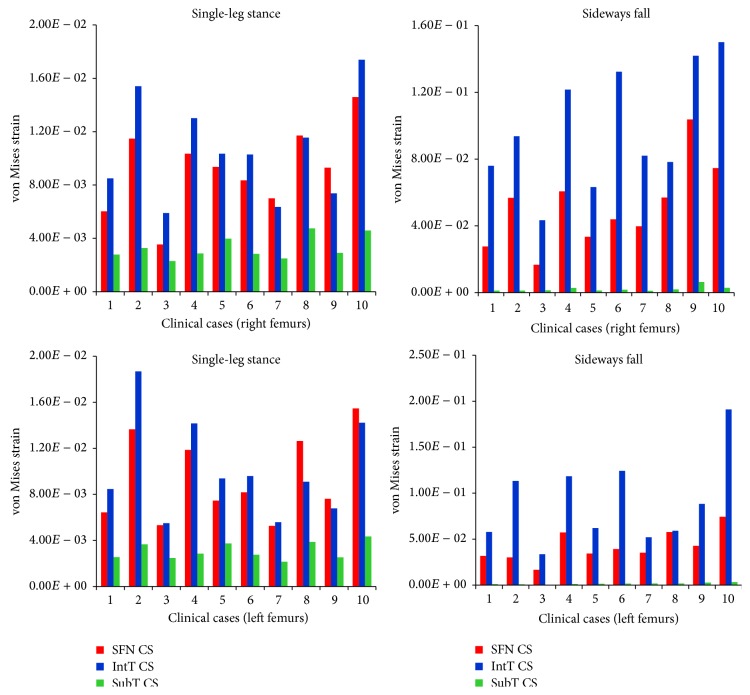
The maximum von Mises strain at the smallest femoral neck cross-section (SFN CS), the intertrochanteric cross-section (IntT CS), and the subtrochanteric cross-section (SubT CS) of right and left femurs of 10 clinical cases during the single-leg stance and the sideways fall.

For the 10 clinical cases (5 females and 5 males, totally 20 right and left femurs), the maximum von Mises stress and strain at the three critical cross-sections of femur during both the single-leg stance and the sideways fall are shown in Figures 8 and 9. It can be observed that, during the sideways fall, the femoral neck and the intertrochanteric region received higher stresses than the subtrochanteric region (Table 4). But during the single-leg stance, the patterns in the stresses are different (Table 3); first, the differences between the stresses over the three regions are much smaller; for some cases, the stresses at the subtrochanteric region are higher than those in the other two regions (Figure 8). Strains in the three regions have similar patterns during both the single-leg stance and the sideways fall (Tables 5 and 6).

### 3.3. Comparison of Hip Fracture Risk at the Three Critical Cross-Sections

**Figure 10:**
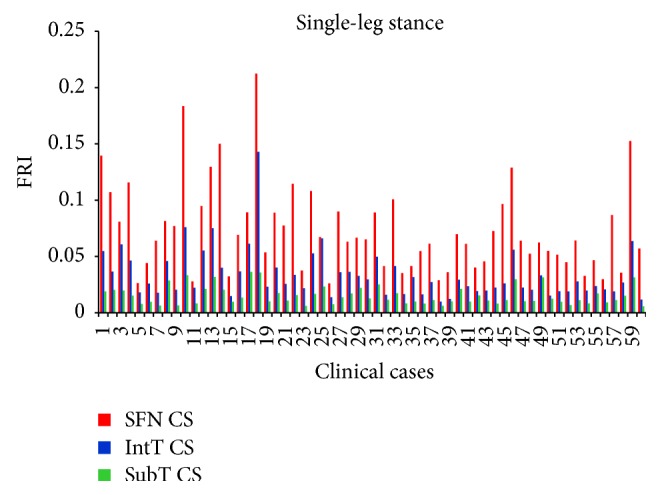
Fracture risk index in single-leg stance configuration.

**Figure 11:**
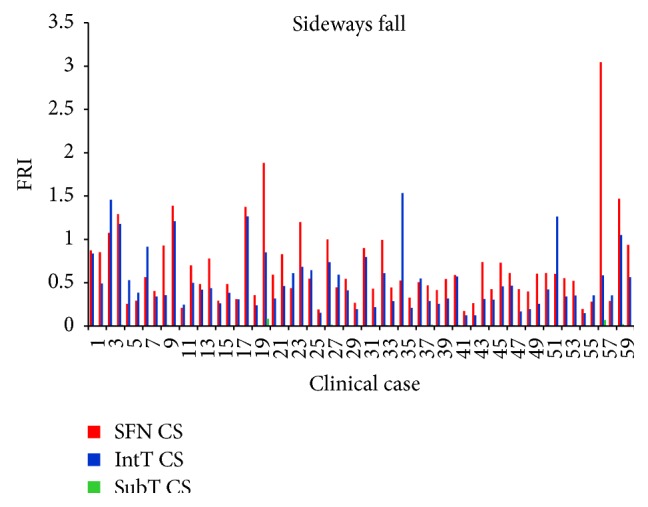
Fracture risk index in sideways fall configuration.

For the 60 clinical cases (30 females and 30 males), hip fracture risk indices based on the strain energy criterion were calculated for the smallest femoral neck, the intertrochanteric, and the subtrochanteric cross-section of femur during the single-leg stance and the sideways fall. The calculated fracture risk indices are shown in Figures 10 and 11.

**Figure 12:**
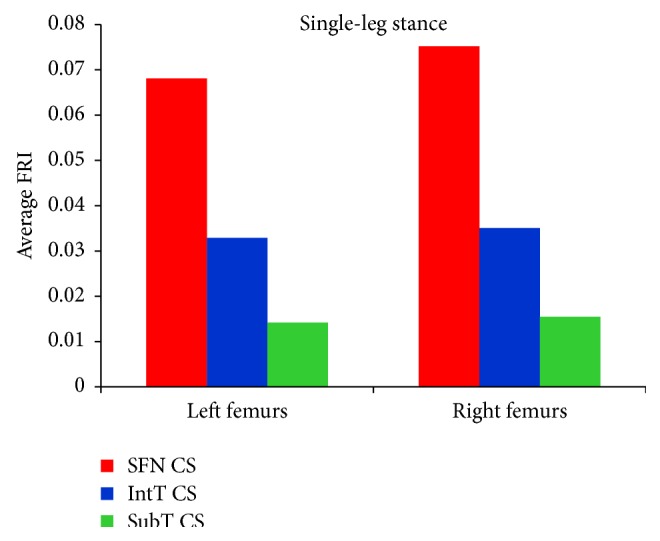
Average FRI at the smallest femoral neck cross-section (SFN CS), the intertrochanteric cross-section (IntT CS), and the subtrochanteric cross-section (SubT CS) of 60 right femurs and 60 left femurs during the single-leg stance.

**Figure 13:**
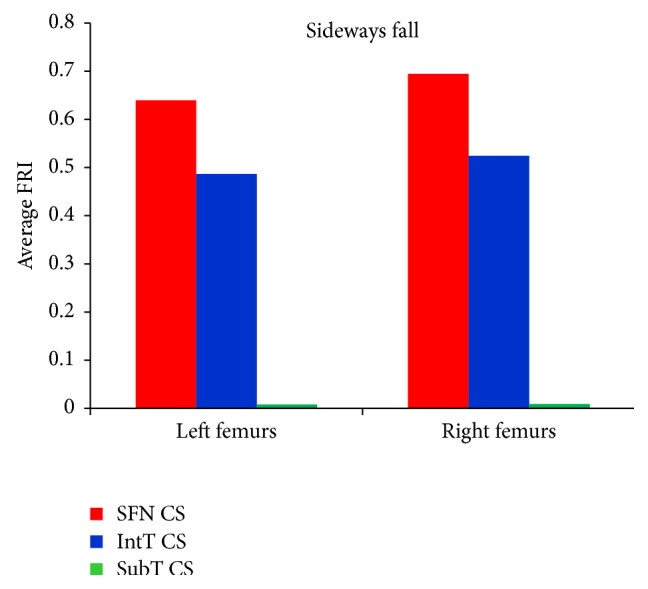
Average FRI at the smallest femoral neck cross-section (SFN CS), the intertrochanteric cross-section (IntT CS), and the subtrochanteric cross-section (SubT CS) of 60 right femurs and 60 left femurs during the sideways fall.

As shown in Tables 7, 8, 9, and 10 and Figures 12 and 13, the average FRI at the smallest femoral neck was higher than those at the intertrochanteric and the subtrochanteric cross-section during both the single-leg stance and sideways fall.

**Figure 14:**
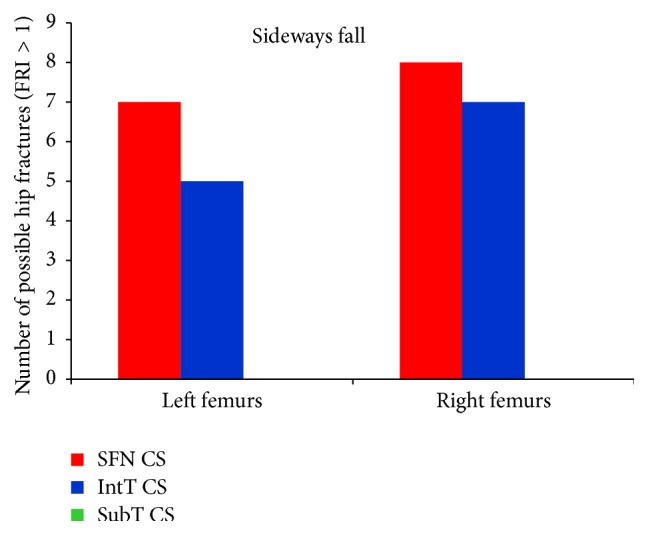
Number of possible hip fractures occurring at the smallest femoral neck cross-section (SFN CS), the intertrochanteric cross-section (IntT CS), and the subtrochanteric cross-section (SubT CS) of 60 right femurs and 60 left femurs during the sideways fall, that is, cases that have the FRI higher than one (FRI > 1) at one of the three critical cross-sections.

For the single-leg stance, FRIs for all cases at the three critical cross-sections are much lower than one (FRI ≪ 1), indicating that the possibility of hip fracture incidence in the single-leg stance was low. For the sideways fall, FRIs of 8 right femurs and 7 left femurs at the smallest femoral neck cross-section and FRIs of 7 right femurs and 5 left femurs at the intertrochanteric cross-section were higher than one (FRI > 1), meaning that there is possibility for fracture occurring in these regions; but the FRIs at the subtrochanteric cross-section in all cases were much lower than one (FRI ≪ 1), indicating that there is lower possibility of fracture in this region.  Figure 14 shows the number of possible fractures at the three critical cross-sections of femur during the sideways fall, that is, the cases that have FRI larger than one (FRI > 1) at one of the three critical cross-sections.

### 3.4. Comparison of Hip Fracture Risk in Women and Men

**Figure 15:**
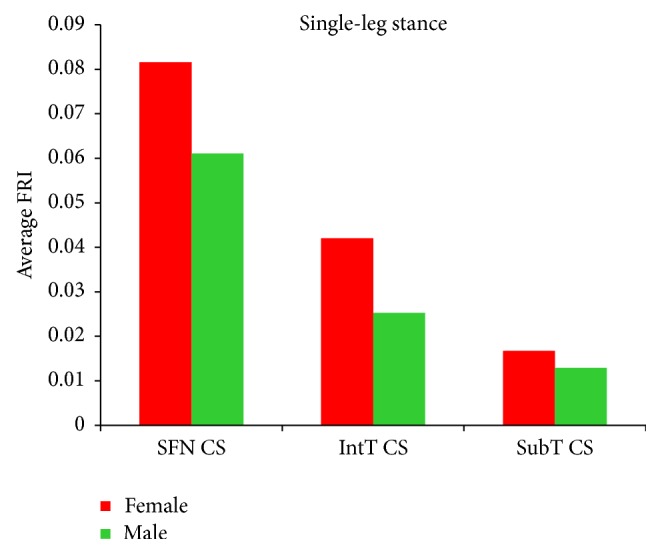
Average FRI at the smallest femoral neck cross-section (SFN CS), the intertrochanteric cross-section (IntT CS), and the subtrochanteric cross-section (SubT CS) of 30 females and 30 males during the single-leg stance.

**Figure 16:**
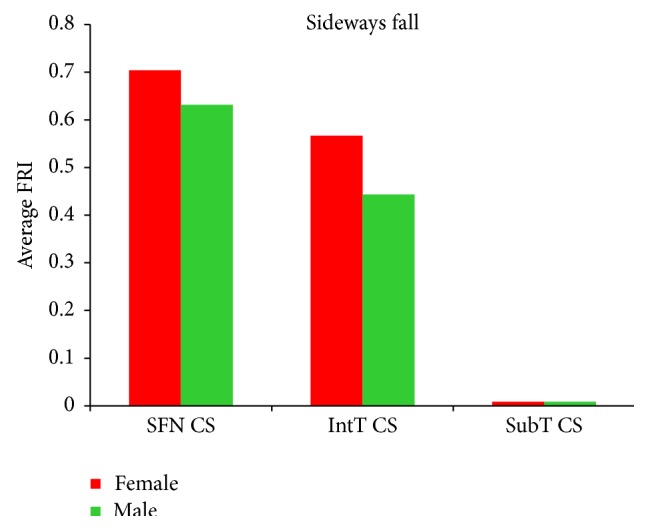
Average FRI at the smallest femoral neck cross-section (SFN CS), the intertrochanteric cross-section (IntT CS), and the subtrochanteric cross-section (SubT CS) of 30 females and 30 males during the sideways fall.

**Figure 17:**
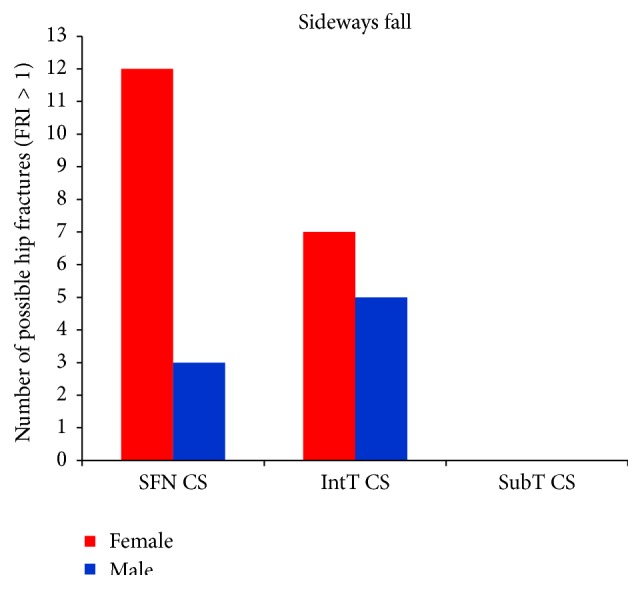
Number of possible hip fractures occurring at the smallest femoral neck cross-section (SFN CS), the intertrochanteric cross-section (IntT CS), and the subtrochanteric cross-section (SubT CS) of 30 females and 30 males during the sideways fall.

In this study, hip fracture risk for the 30 females and 30 males was assessed. The average hip fracture risk of females was generally higher than that of males. As it can be seen from Tables 11 [Table tab12]–13 and Figures 15 and 16, the average FRI at the smallest femoral neck cross-section, the intertrochanteric cross-section, and the subtrochanteric cross-section of the 30 females is higher than that of the 30 males for both the single-leg stance and the sideways fall.  Figure 17 shows the number of possible hip fractures in women and men during the sideways fall for the studied cases.

## 4. Discussion

Hip fracture may occur anywhere from the articular cartilage of the hip joint to the femur shaft [48]. Not only the location of the fracture types but also the etiology differs. It was reported that women with the intertrochanteric fracture have significantly lower BMD than those with the femoral neck fracture [49–51]. On the other hand, the femoral neck fracture may not be mainly attributed to low BMD but may be related to external causes such as sideways fall [52]. Femoral neck and intertrochanteric fractures are often the result of falls from standing height and impact onto the greater trochanter, particularly for the elderly. The subtrochanteric fractures, on the other hand, are typically the result of high energy impacts such as motor vehicle accidents and falls from a height [53].

The selection of bone failure criterion is challenging. In literatures, stress and strain based failure criteria such as the von Mises stress and strain criteria and the maximum principle stress and strain criteria were commonly used to assess hip fracture risk. To the best of our knowledge, the strain energy based failure criterion has not been used yet for hip fracture risk assessment. Whereas the cancellous bone failure is in the form of buckling and deformation (strain intensity) and the cortical bone failure is related to its local cracking (stress intensity), strain energy failure criterion, which is a combination of both stress and strain intensities, is theoretically more reasonable than other failure criteria for hip fracture risk assessment. The differences between the strains in the three critical regions of femur during both the single-leg stance and the sideways fall (Figure 9, Tables 5 and 6) are much higher than the differences between the corresponding stresses (Figure 8, Tables 3 and 4), indicating that bone failure is more sensitive to the strains because of its fragility property and the effects of strains should also be considered in bone fracture risk assessment.

Results of this study show that the femoral neck and the intertrochanteric region have higher fracture risk than the subtrochanteric region (Figures 12 and 13 and Tables 7–10), which is consistent with the fact that the femoral neck and the intertrochanteric region have a larger proportion of cancellous bone than the subtrochanteric region; and the cancellous bone is generally weaker than the cortical bone. Therefore, hip fracture is most likely to initiate first at the femoral neck and then in the intertrochanteric region or in the subtrochanteric region. For all subjects, there is very low hip fracture risk during the single-leg stance (FRI < 1). Among all the cases, during the sideways fall, 15 femurs at the femoral neck and 12 femurs at the intertrochanteric region have FRI higher than one (FRI > 1), while there is very low fracture risk at the subtrochanteric region for all the subjects (FRI < 1) (Figure 14). Our findings have a good agreement with the previous clinical observations. According to the clinical observations, the femoral neck is the most common location for a hip fracture, accounting for 45% to 53% of hip fractures; the intertrochanteric fractures account for approximately 38% to 50% of all hip fractures; and the subtrochanteric fractures are less common than the femoral neck and intertrochanteric fractures, accounting for approximately 5% to 15% of hip fractures [54, 55]. Also it was observed that, in old people with age between 65 and 99, femoral neck and intertrochanteric fractures occurred with approximately the same frequency [56]. Data on 176 geriatric patients with hip fractures showed that 59% were the intertrochanteric fractures while the rest (41%) were the intracapsular neck fractures [57]. In another study by Michelson et al. [43], it was observed that 37% of hip fractures are in the femoral neck, 49% are intertrochanteric, and 14% are subtrochanteric. Therefore, the femoral neck and the intertrochanteric region are more disposed to fracture than the subtrochanteric region.

Both the fracture risk level and the potential fracture location are patient-dependent and depend on BMD and other conditions that have not been studied in this paper. We have found that women generally have lower bone strength than men and thus are exposed to higher hip fracture risk during both the single-leg stance and the sideways fall (Figures 15 and 16 and Tables 11–13). Based on the calculated FRI, in the 30 women potentially there are 12 femoral neck fractures and 7 intertrochanteric fractures, while for the 30 men there are only 3 potential femoral neck and 5 potential intertrochanteric fractures (Figure 17). Our finding is consistent with other studies that have found that hip fracture risk in women is generally higher than in men. The results of a worldwide study by Dhanwal et al. [58] on incidence and epidemiology of hip fractures in Asia, Africa, Europe, Latin America, North America, and Oceania show that women are more disposed to the hip fracture risk than men in different countries over the world. In the study by Jacobsen et al. [59], the age-adjusted hip fracture incidence rates in white US males and females were 4.3 and 8.1 per 1,000 per year, respectively. Higher hip fracture risk in women may be related to their lower BMD.

## 5. Conclusion

A reliable methodology to assess hip fracture risk in individuals is crucially important for preventing hip fracture and initiating a treatment. The purpose of this study is to propose a more effective hip fracture risk index that is based on the strain energy failure criterion, and it is able to better describe bone failure mechanism and microstructure. The proposed fracture risk index can predict not only the fracture risk level, but also the potential fracture location. The results of this study showed that there is a very low hip fracture risk during the single-leg stance, while, during the sideways fall, there is a high fracture risk at the femoral neck and the intertrochanteric region, compared to the subtrochanteric region. Based on the results obtained from this study, women are more prone to hip fracture than men. The procedure described in this study can be implemented into computer programming and used in hip fracture prevention and monitoring of osteoporosis treatments in the elderly. The method may also help design more effective hip protectors by providing feedback information such as stresses/strains in the hip for adjusting the design parameters. The main limitation of this study is that no experiment has been conducted to validate the predicted fracture risk levels and potential fracture locations, which has been set as future work.

## Figures and Tables

**Table 1:** Statistical information of the 60 clinical cases.

Gender		Age (years)	Height (cm)	Body weight (kg)	BMI (kg/m^2^)
Female	Range	50–82	149–174.7	51.7–110.9	21.01–43.36
	Average	65	163.92	77.58	28.81

Male	Range	50–78	163.4–193.2	61.5–126.6	18.83–40.96
	Average	64.71	175.74	86.22	27.9

**Table 2:** Femoral neck FRI obtained with different number of integration points.

Case number	FRI		Relative error (%)
	3 integration points	7 integration points	
1	0.239	0.2416	1.07
2	0.6898	0.6975	1.1
3	0.2966	0.2976	0.33
4	0.8885	0.899	1.16
5	1.1482	1.1701	1.87

**Table 3:** Average maximum von Mises stress (MPa) at the smallest femoral neck cross-section (SFN CS), the intertrochanteric cross-section (IntT CS), and the subtrochanteric cross-section (SubT CS) of right and left femurs of 10 clinical cases during the single-leg stance.

Maximum von Mises stress (MPa)						
		Right femurs			Left femurs	
	SFN CS	IntT CS	SubT CS	SFN CS	IntT CS	SubT CS
Range	21.7–49.96	22.23–45.37	26.93–52.47	19.56–52.38	23.55–47.8	27.09–43.04
Average	33.63	32.97	37.89	32.93	32.41	35.84

**Table 4:** Average maximum von Mises stress (MPa) at the smallest femoral neck cross-section (SFN CS), the intertrochanteric cross-section (IntT CS), and the subtrochanteric cross-section (SubT CS) of right and left femurs of 10 clinical cases during the sideways fall.

Maximum von Mises stress (MPa)						
		Right femurs			Left femurs	
	SFN CS	IntT CS	SubT CS	SFN CS	IntT CS	SubT CS
Range	26.69–148.53	21.57–74.3	9.8–70.63	22.78–69.97	16.2–60.3	6.73–33.2
Average	57.22	40.74	27.08	46.52	33.48	18.66

**Table 5:** Average maximum von Mises strain at the smallest femoral neck cross-section (SFN CS), the intertrochanteric cross-section (IntT CS), and the subtrochanteric cross-section (SubT CS) of right and left femurs of 10 clinical cases during the single-leg stance.

		Range	Average
	Right femurs		
	SFN CS	3.54*E* − 03–1.46*E* − 02	9.15*E* − 03
	IntT CS	5.89*E* − 03–1.74*E* − 02	1.08*E* − 02
Maximum von Mises strain	SubT CS	2.3*E* − 03–4.75*E* − 03	3.32*E* − 03
	Left femurs		
	SFN CS	5.25*E* − 03–1.55*E* − 02	9.55*E* − 03
	IntT CS	5.49*E* − 03–1.87*E* − 02	1.05*E* − 02
	SubT CS	2.14*E* − 03–4.34*E* − 03	3.11*E* − 03

**Table 6:** Average maximum von Mises strain at the smallest femoral neck cross-section (SFN CS), the intertrochanteric cross-section (IntT CS), and the subtrochanteric cross-section (SubT CS) of right and left femurs of 10 clinical cases during the sideways fall.

		Range	Average
	Right femurs		
	SFN CS	1.67*E* − 02–1.04*E* − 01	5.29*E* − 02
	IntT CS	4.34*E* − 02–1.50*E* − 01	9.80*E* − 02
Maximum von Mises strain	SubT CS	12*E* − 03–6.38*E* − 03	2.44*E* − 03
	Left femurs		
	SFN CS	1.67*E* − 02–7.43*E* − 02	4.26*E* − 02
	IntT CS	3.35*E* − 02–1.91*E* − 01	9.37*E* − 02
	SubT CS	5.08*E* − 04–3.31*E* − 03	1.74*E* − 03

**Table 7:** Average FRI at the three critical cross-sections of 60 right femurs during the single-leg stance.

		FRI	
	Smallest femoral neck cross-section	Intertrochanteric cross-section	Subtrochanteric cross-section
Range	0.0261–0.2124	0.0099–0.143	0.0058–0.0363
Average	0.0752	0.0351	0.0155

**Table 8:** Average FRI at the three critical cross-sections of 60 left femurs during the single-leg stance.

		FRI	
	Smallest femoral neck cross-section	Intertrochanteric cross-section	Subtrochanteric cross-section
Range	0.023–0.1936	0.0095–0.1078	0.0037–0.0337
Average	0.0681	0.0329	0.0142

**Table 9:** Average FRI at the three critical cross-sections of 60 right femurs during the sideways fall.

		FRI	
	Smallest femoral neck cross-section	Intertrochanteric cross-section	Subtrochanteric cross-section
Range	0.1725–3.0448	0.1226–1.534	0.0004–0.0812
Average	0.6944	0.5245	0.0091

**Table 10:** Average FRI at the three critical cross-sections of 60 left femurs during the sideways fall.

		FRI	
	Smallest femoral neck cross-section	Intertrochanteric cross-section	Subtrochanteric cross-section
Range	0.1599–1.8301	0.116–1.5493	0.0004–0.0585
Average	0.6395	0.4864	0.0083

**Table 11:** Average FRI at the smallest femoral neck cross-section of 30 females and 30 males during the single-leg stance and the sideways fall.

FRI at the smallest femoral neck cross-section				
	Single-leg stance		Sideways fall	
	Female	Male	Female	Male
Range	0.023–0.2124	0.0231–0.1628	0.1826–1.8809	0.1599–3.0448
Average	0.0816	0.0611	0.7035	0.6315

**Table 12 tab12:** Average FRI at the intertrochanteric cross-section of 30 females and 30 males during the single-leg stance and the sideways fall.

FRI at the intertrochanteric cross-section				
	Single-leg stance		Sideways fall	
	Female	Male	Female	Male
Range	0.0138–0.143	0.0095–0.0637	0.1398–1.4572	0.116–1.5493
Average	0.042	0.0253	0.5666	0.4433

**Table 13:** Average FRI at the subtrochanteric cross-section of 30 females and 30 males during the single-leg stance and the sideways fall.

FRI at the subtrochanteric cross-section				
	Single-leg stance		Sideways fall	
	Female	Male	Female	Male
Range	0.0037–0.0363	0.0053–0.0315	0.0004–0.0812	0.0004–0.0694
Average	0.0167	0.0129	0.0087	0.0088
