# Corrigendum to “Assessment of Hip Fracture Risk Using Cross-Section Strain Energy Determined by QCT-Based Finite Element Modeling”

**DOI:** 10.1155/2017/4791706

**Published:** 2017-07-03

**Authors:** Hossein Kheirollahi, Yunhua Luo

**Affiliations:** ^1^Department of Mechanical Engineering, Faculty of Engineering, University of Manitoba, Winnipeg, MB, Canada R3T 5V6; ^2^Department of Anatomy, Southern Medical University, Guangzhou 510515, China

In the article titled “Assessment of Hip Fracture Risk Using Cross-Section Strain Energy Determined by QCT-Based Finite Element Modeling” [1], there was an error regarding the FRAX® tool, which should be clarified as follows:

The article notes: “Fracture Risk Assessment Tool (FRAX) is a tool to evaluate an individual's fracture probability in the next 10 years, adopted by the WHO in 2008 [7].” However, the World Health Organization (WHO) did not develop, test or endorse the FRAX® tool or its recommendations [2]. The metabolic bone disease unit at the University of Sheffield that developed FRAX® was a WHO Collaborating Centre from 1991 to 2010, but treatment guidelines must undergo a formal process before they can be endorsed by the WHO.
